# The assessment of psychometric properties for the subjective wellbeing-5 dimensions (SWB-5D) questionnaire in the general Dutch population

**DOI:** 10.1007/s11136-022-03234-8

**Published:** 2022-08-20

**Authors:** Heleen N. Haspels, Marieke de Vries, M. Elske van den Akker-van Marle

**Affiliations:** 1 Department of Biomedical Data Science, Section Medical Decision Making, Leiden University Medical Center, PO Box 9600, 2300 RC Leiden, The Netherlands; 2 Institute for Computing and Information Sciences (iCIS), Radboud University, Nijmegen, The Netherlands

**Keywords:** Quality of life, Economic evaluations, Construct validity, Concurrent validity, Subjective wellbeing

## Abstract

**Purpose:**

Financial resources for health care are limited, so assessment of intervention effectiveness in terms of health in relation to its costs is important. Measuring health outcomes in cost-effectiveness analyses is usually done by health-related quality of life measures, like the EQ-5D. However, over the past decade, innovations on the conceptual level of health have evolved and novel approaches are rising such as the capability approach, subjective wellbeing, and Positive Health. This study assesses the psychometric properties of the subjective wellbeing-5 dimension (SWB-5D) outcome measure.

**Methods:**

A quantitative, cross-sectional study design was used to determine the concurrent and construct (convergent and known group) validity for the SWB-5D. Concurrent and convergent validity were estimated as correlations between the SWB-5D and the Dutch version of the EQ-5D, ICECAP-A, and PH-17. Assessment of known-groups validity was based on the variables illness, education, and the overall happiness (Cantril Ladder) and overall health scale (EQ-5D VAS).

**Results:**

A representative sample of 1016 respondents of the Dutch population completed an online questionnaire. The SWB-5D showed reasonable concurrent validity and showed good convergent and known-group validity. The SWB-5D had a lower ceiling effect compared to the EQ-5D and ICECAP-A.

**Conclusion:**

Compared to traditional health measurement approaches, novel approaches are more focused on the mental and social pillars of health. The SWB-5D shows psychometric feasibility of comprehensive measurement of health, as indicated by a range of validity measures in a large representative sample of the Dutch population.

**Supplementary Information:**

The online version contains supplementary material available at 10.1007/s11136-022-03234-8.

## Introduction

Financial resources for health care are inevitably limited, so assessment of intervention effectiveness in relation to its costs is important to support the distribution of the scarce resources. Cost-effectiveness analyses (CEAs) theoretically enable direct comparison of the costs of obtaining health outcomes between different healthcare interventions. Health outcomes have increasingly been assessed using a multi-attribute utility instrument (MAUI) [1]: a generic health-related questionnaire about the health-related quality of life (HRQoL) combined with a (country specific) accompanying formula or set of weights for converting responses into utility scores.

During the development of the MAUIs, different conceptual models of health have been used to measure health, based on the current World Health Organization’s (WHO) definition of health, first formulated in 1948. It describes health as “a state of complete positive physical, mental and social wellbeing and not merely the absence of disease of infirmity” [2]. Thereby, three building blocks of health are defined: physical, mental, and social. HRQoL is most commonly measured with the EQ-5D instrument [3].

However, there is a rising critique as the frequently used HRQoL measures seem to employ a narrow view on quality of life, with a predominant focus on physical impairments rather than mental and social wellbeing [4, 5]. This is important in the context of economic evaluations when interventions do not (only) affect physical functioning but also the other factors of quality of life, for example in the context of social care, mental health care [6], chronic care, and elderly care [7]. Currently, the effectiveness of interventions for these patient groups might be underestimated, which could ultimately lead to unwarranted underallocation of health care resources for these interventions.

The subjective wellbeing-5 dimensions (SWB-5D) is a new measurement tool and is intended to be better suited to assess quality of life comprehensively [8] (Pietersma, de Vries, & van den Akker-van, 2014). The intention was that the SWB-5D would be able to detect important effects of health interventions in situations that are more care related than cure related, such as, end-of-life care, informal care, in vitro fertilization, and chronic diseases.

The SWB-5D has been developed in two phases: (1) using a Delphi procedure in different stakeholdergroups to have a good understanding of what stakeholders perceive as important for HR-SWB [9] and (2) an exploratory factor analysis to investigate whether the identified domains in de Delphi can be summarized in a limited number of underlying factors [8]. This resulted in the identification of five domains, physical indepence, positive affect, negative affect, autonomy, and personal growth, each of which is represented by one item in the SWB-5D, see Online Resource 1. The next step is establishing the validity of the SWB-5D, which is vital in order to confidently use this instrument.

The aim of the current study is to assess the concurrent and construct (convergent and known group) validity and interpretability of the SWB-5D.

## Methods

### Design and participants

A quantitative approach was used to assess the psychometric properties of the SWB-5D by studying a representative sample of the Dutch population. This consisted of a cross-sectional design using a Web-based questionnaire which included the SWB-5D and other health-related scales. The participants were recruited by means of quota sampling in June 2020 by a research market agency, with high-quality requirements and a ISO Certification. The sample was representative of the general Dutch population in terms of demographic variables (gender, age, education, and residential area). An invitation email was send to 1540 members with a brief description of the study. Individuals willing to participate (*N* = 1016, 66% of the invited panellists) received points in exchange for their participation (if enough points are collected they were given a gift coupon). Participants were informed about the study, asked for informed consent, and anonymized prior to the study. To avoid any missing data, it was not possible to skip any question. Participants were allowed to complete the questionnaire with breaks.

An independent medical ethics committee evaluated the study and confirmed it did not fall within the Medical Research Act, waiving the need for ethical approval as this study did not provide any intervention to participants, and the questionnaires were not perceived to be invasive to them (METC-LDD 19-035).

### Measurements

#### Demographics

Extracted information on demographics was (1) age in years, (2) current living region or province, (3) gender, (4) chronic illness (yes/no), and (5) highest completed education level with nine categories (ranging from “no education” to “university”) that were later transformed to lower, middle, and higher education.

#### SWB-5D

The SWB-5D instrument consists of five items representing five dimensions: physical independence, happiness, loneliness, autonomy, and personal growth (Online Resource 1**)** [8]. One question for each of the dimensions has been developed and the answers can be given using a 5-point Likert Scale ranging from totally disagree to strongly agree. Negatively worded items were reverse coded. The total score was obtained by summing the mean scores of the five dimensions and ranged between five and 25, with higher scores indicating higher wellbeing.

#### Validation scales

To test the concurrent validity of the SWB-5D, the relationship between the 5-item SWB model and other health-related scales was tested. The chosen validations scales are (1) the EQ-5D since this is the most commonly used measurement tool in economic evaluations, (2) the ICECAP-A since this tool is developed for use in economic evaluations of health and social care interventions by focussing on wellbeing defined in a broader sense rather than health, (3) the PH-17 measurement tool which aims to capture the concept Positive Health: health as the ability to adapt and to self manage, in the face of social, physical, and emotional challenges and (4) Cantril ladder, an overall rating scale for wellbeing.

#### EQ-5D

The EQ-5D-5L was used to measure different domains of health and consists of five items representing five dimensions: mobility, self-care, usual activity, pain/discomfort, and anxiety/depression with five levels for each item ranging from “no problems” to “extreme problems” [3]. Utility scores are derived from preferences of the Dutch citizens [10] ranging from 1 (perfect health) to − 0.446 (worse than death) and anchored at 0 (death). The EQ-5D also contains a visual analogue scale (EQ-VAS) which records subject’s self-reported health on a vertical scale ranging from 0 (worst health you can imagine) to 100 (best health you can imagine).

#### ICECAP-A

The ICEpop CAPability measure for Adults (ICECAP-A) is designed to measure people’s capability (what an individual can do) rather than function (what they actually do) to highlight the importance of freedom to choose [11]. The measures consist of 5 items representing five dimensions: stability, attachment, autonomy, achievement, and enjoyment. Four response levels were defined for respondents to describe their level of ability to achieve these wellbeing states ranging from unable/cannot to completely/all/a lot. ICECAP-A scores were transformed into capability values using the Dutch tariff ranging from 1 (full capability) to 0 (no capability) [12].

#### PH-17

The Postive Health (PH)-17 has a six-dimension structure comprising physical fitness, mental functions, future perspective, contentment, social relations, and daily life-management [13]. For each dimension, two or three items are proposed on an 11-point Likert scale, ranging from totally disagree to totally agree. Scores for the six dimensions were computed as the mean score on the statements per dimensions. The total score was obtained by summing the mean scores of the six dimensions and ranged between 11 and 66, with higher scores indicating better health.

#### Cantril ladder

Cantril Ladder uses a vertical, visual analogue scale in which respondents can indicate to what extent they had the best possible life [14]. An 11-rung ladder was used ranging from 0 (worst possible life) to 10 (best possible life).

### Statistical analysis

The psychometric testing of the SWB-5D in this study consisted of assessing the concurrent and construct (convergent and known group) validity and interpretability of the SWB-5D (see graphical presentation in Online Resource 2).

#### Concurrent validity

Data were analysed using IBM SPSS version 25. To explore the concurrent validity, multivariable regression analysis, using ordinary least-squares regression, was used. We assessed the degree to which the SWB-5D items were explained by the items of the EQ-5D, ICECAP-A, and PH-17 and the overall measures for health and wellbeing, respectively, the EQ-VAS and Cantril Ladder. The overall strength of the statistical relationships between SWB-5D and the other domains (*R*^2^) provided a quantification of how well the SWB-5D correlated with other scales. The *R*^2^ coefficient is a statistic on a 0–1 scale, where “1” means that the SWB-5D variation can be fully explained by other items and “0” represents no relationship between other measurement tools and the SWB-5D.

#### Construct validity

To evaluate construct validity of the SWB-5D, convergent validity and known-groups validity were investigated.

##### Convergent validity

For the total scores and item comparison, correlations were assessed using Spearman’s rank correlation coefficients. Correlation sizes below 0.2 were considered absent, those from 0.2 to less than 0.35 were considered weak, those from 0.35 to less than 0.50 were considered moderate, and those of 0.5 or greater were considered very strong [15]. Differences of *p* < 0.05 were considered to be significant. To correct for type 1 error rate (false-positive correlations), Holm-Bonferroni correction for multiple testing was applied [16]. Instead of dividing the level of every test by the number of all tests, the Bonferroni-Holm procedure orders the test results from the smallest to the highest *p* value and adjusts the α or *p* values sequentially. Prior to examining the data, a list of hypotheses was designed (Online Resource 2)**.** At least 75% of these hypotheses should be confirmed to indicate good convergent validity [17]. Convergent validity assesses the extent of correlation between instruments intended to measure similar or overlapping constructs, in our case quality of life [18]. The convergent validity of the SWB-5D was determined by calculating Spearman rank correlations to test their association with the health (EQ-5D and EQ-VAS), capability (ICECAP-A), and wellbeing (Cantril Ladder) measures. A list of all hypotheses on convergent validity and a visualization of the overlapping concepts tested can be found in Online Resource 2.

Hypothesis 1 (H1) concerned the correlation between SWB-5D sum score and utility score of the EQ-5D. Both questionnaires aim to measure quality of life, albeit with a different approach. However, we would expect that they correlate. Four out of the five questions of EQ-5D are based on the physical pillar of health. Therefore we hypothesized that these items correlate with the physical independence dimension of the SWB-5D (H2-5). Loneliness (SWB-5D) and anxiety/depression (EQ-5D) were expected to correlate because even though these concepts differ, both should indicate “no happy feelings” (H6). Furthermore, we expected that high happiness scores (SWB-5D) would correlate with low anxiety/depression (EQ-5D) scores (H7). Similar to hypothesis 1, we expect the SWB-5D sum score to correlate with the EQ-VAS score (H8).

We expected that sum score of the SWB-5D correlates with the ICECAP-A utility score (H9). Having problems concerning physical independence as captured by the SWB-5D might involve more dependency on others, which would be reflected in lower autonomy scores on the ICECAP-A (H10). Furthermore, we expected that happiness and loneliness were related to enjoyment and attachment (H11–14). Both questionnaires include a domain of autonomy, for which we expected that they would relate to each other (H15). Moreover, we expected that personal growth (SWB-5D) and achievement (ICECAP-A) would be related (H16). Finally, the SWB-5D sum score was hypothesized to correlate with the Cantril Ladder score (H17).

##### Known-groups validity

Known-groups validity was defined as the ability of the questionnaire to discriminate between subgroups. This was assessed by comparing differences in SWB-5D scores as a function of EQ-VAS score (below or above 65), Cantril Ladder score (below or above 7), illness (present or absent), and education (low, medium, or high). We expected higher SWB-5D scores for respondents with higher self-reported health/happiness scales (EQ-VAS and Cantril Ladder) and no illnesses. Based on earlier research, we also expected a difference between educational groups with lower reported health status for respondents with lower education [19]. Details on the hypotheses can be found in Online Resource 2. Since the SWB-5D scores did not follow a normal distribution, associations between the SWB-5D scores and selected variables were tested using a Mann–Whitney *U* test for two group comparisons and a Kruskal Wallis H test for comparisons between three groups. Again, Holm-Bonferroni correction for multiple testing was applied. In addition, the standard error of the mean (SEM) was calculated, which allows us to identify whether outcome changes between measures can be attributed to a real modification. The SEM is equal to the square root of the error variance: SEM = ${{\sqrt{\sigma } }^{2}}_{\text{error}}$ [20, 21].

#### Interpretability

Interpretability was analysed by determining the ceiling effects [22]. The occurrence of ceiling effects was estimated in order to investigate whether the SBW-5D scale covered the full variance in the respondent group [23]. Ceiling effects are considered present if a large population of respondents uses the highest rating, respectively. The threshold was considered to be reachted if more than 15% of the respondents achieved the maximum scores [24].

## Results

### Participants

The questionnaire was completed by 1016 participants, the descriptive statistics of the study sample are presented in Table 1. The sample’s demographic characteristics were fairly similar to that of the Dutch population (49% women; age: from 18 until 39 = 29.6%, from 40 until 65 = 43.5%, older than 65 = 27%; educational level: low = 28.7%, medium = 42.6%, high = 28.6%). Approximately, 53% of the respondents reported to have one or more chronic disease(s).

**Table 1:** Characteristics of participants

Variable	Category	Frequency (%)/mean (SD)
Sex	Female	49.6%
	Male	50.4%
Age (years)	18–39	29.6%
	40–65	43.5%
	65+	27.0%
Education	Lower	28.7%
	General	42.6%
	High	28.6%
Chronic disease	Present	53.2%
	Absent	46.8%
SWB-5D	Scale	19.6 (2.9)
EQ-5D	Index score	0.669 (0.309)
EQ-VAS	Scale	74.2 (17.3)
ICECAP-A	Capability value	0.61 (0.216)
PH-17	Scale	51 (6.9)
Cantrils ladder	Scale	7.45 (1.55)

The score range of; SWB-5D is between 5 (low wellbeing) and 25 (high wellbeing), EQ-5D is between -0.446 (worse than death) and 1 (perfect health), EQ-VAS between 0 and 100, ICECAP-A between 0 and 1, PH-17 between 11 (worst health) and 66 (best health) and Cantril Ladder between 0 (worst possible life) to 10 (best possible life)

*SWB-5D* subjective wellbeing-5 dimensions, *EQ-5D* EuroQol five-dimensional questionnaire, *EQ-VAS* EuroQol Visual Analogue Scale, *ICECAP-A* ICEpop CAPability Adult

### Concurrent validity

The relationship between the SWB-5D scores and items of the other scales showed a diverse picture, see Table 2. While the SWB-5D explained most of the variance of items such as “Contentment” (PH-17, *R*^2^ = 0.637), “Physical fitness” (PH-17, *R*^2^ = 0.478), “Future perspective” (PH-17, *R*^2^ = 0.448), “Mobility” (EQ-5D, *R*^2^ = 0.403), “Social relations” (PH-17, *R*^2^ = 0.401) and the overall wellbeing measure Cantril Ladder (*R*^2^ = 0.427), it was less related to items such as “Autonomy” (ICECAP-A, *R*^2^ = 0.098), “Self-care” (EQ-5D, *R*^2^ = 0.119), “Stability” (ICECAP-A, *R*^2^ = 0.176), and “Achievement” (ICECAP-A, *R*^2^ = 0.199).

**Table 2:** Multivariable regression analysis between the five items of SWB-5D and health-related measurement tools

Scale	Domain	Standardized loadings (*β*) on SWB-5D dimensions					*R*^2^
		Physical independence	Happiness	Loneliness	Autonomy	Personal growth	
EQ-5D	Mobility	0.633***	− 0.007	0.018	0.002	0.040	0.403
	Self-care	0.331***	0.051	0.010	0.027	0.001	0.119
	Usual activities	0.427***	0.170***	− 0.078*	0.016	0.004	0.283
	Pain/discomfort	0.451***	0.085*	− 0.012	− 0.047	− 0.012	0.223
	Anxiety/depression	− 0.020	0.345***	− 0.311***	0.045	− 0.053	0.329
EQ-VAS		0.371***	0.283***	− 0.09**	0.003	0.028	0.329
Cantrils ladder		0.138***	0.494***	− 0.163***	0.019	− 0.002	0.427
ICECAP-A	Stability	0.067*	0.269***	− 0.115*	0.129***	− 0.014	0.176
	Attachment	− 0.022	0.309***	− 0.210***	0.013	0.080**	0.229
	Autonomy	0.169***	0.091*	− 0.126***	0.077*	0.026	0.098
	Achievement	0.135***	0.255***	− 0.101**	0.019	0.144***	0.199
	Enjoyment	0.036	0.393***	− 0.154***	0.073**	0.092**	0.318
PH-17	Physical fitness	0.565***	0.231***	− 0.049	0.003	0.034	0.478
	Mental functions	0.181***	0.251***	− 0.127***	0.013	0.108***	0.216
	Future perspective	0.091***	0.378***	− 0.153***	0.020	0.305***	0.448
	Contentment	0.124***	0.598***	− 0.198***	0.046*	0.049*	0.637
	Social relations	0.034	0.371***	− 0.260***	0.053*	0.136***	0.401
	Daily life-management	0.210***	0.242***	− 0.153***	0.157***	0.078**	0.300

*SWB-5D* subjective wellbeing-5 dimensions, *EQ-5D* EuroQol five-dimensional questionnaire, *EQ-VAS* EuroQol Visual Analogue Scale, *ICECAP-A* ICEpop CAPability Adult

*p < 0.05, **p < 0.01, ***p < 0.001

As presented in Table 2, each of the five factors of the SWB-5D was important to explain variance across the validation scales. For example, “Physical independence” was an important predictor of “Mobility” (EQ-5D, *β* = 0.633, *p* < 0.001) and “Physical fitness” (PH-17, *β* = 0.565, *p* < 0.001). “Happiness” was a predictor of “contentment” (PH-17, *β* = 0.598, *p* < 0.001) and Cantril Ladder (*β* = 0.494, *p* < 0.001); “Loneliness” a predictor of “Anxiety/depression” (EQ-5D, *β* = − 0.311, *p* < 0.001) and “Social relations” (PH-17, *β* = − 0.260, *p* < 0.001); “Autonomy” was a predictor of “Daily life management” (PH-17, *β* = 0.157, *p* < 0.001) and “Stability” (ICECAP-A, *β* = 0.129, *p* < 0.001), and “Personal growth” was a predictor of “Future perspective” (PH-17, *β* = 0.305, *p* < 0.001). The factor “Autonomy” showed the weakest association with the validation scales (range *β* = − 0.047–0.157), followed by “Personal growth” (range *β* = − 0.053–0.305) and “Loneliness” (range *β* = − 0.311–0.018). “Physical independence” showed the strongest association with the validation scales (range *β* = − 0.022–0.633), followed by “Happiness” (range *β* = − 0.007–0.598).

### Construct validity

Seventeen hypotheses were tested to investigate the convergent validity of the SWB-5D (Online Resource 2). The correlation matrix between the SWB-5D, EQ-5D, and ICECAP-A can be found in Online Resource 3. A strong Spearman correlation was found between the SWB-5D sum score and the ICECAP-A index score (*R* = 0.520**), and a moderate correlation was found between the SWB-5D sum score and the EQ-5D index score (*R* = 0.480**). Fourteen out of the seventeen (78%) hypotheses were confirmed, which indicated a good convergent validity of the SWB-5D (see Table 3).

**Table 3:** Results on convergent validity hypotheses for SWB-5D

Hypothesis	SWB-5D scale	EQ-5D scale	Spearman’s rho	Confirmed
H1	Sum score	Index score	0.480**	Yes
H2	Physical independence	Mobility	0.62**	Yes
H3	Physical independence	Self-care	0.331**	Yes
H4	Physical independence	Usual activity	0.484**	Yes
H5	Physical independence	Pain/discomfort	0.444**	Yes
H6	Loneliness	Anxiety	− 0.477**	Yes
H7	Happiness	Anxiety	0.443**	Yes
H8	Sum score	VAS score	0.529**	Yes

Hypothesis	SWB-5D scale	ICECAP-A scale	Spearman’s rho	Confirmed
H9	Sum score	Index score	0.520**	Yes
H10	Physical independence	Autonomy	0.220**	No
H11	Happiness	Enjoyment	0.511**	Yes
H12	Happiness	Attachment	0.430**	Yes
H13	Loneliness	Enjoyment	− 0.396**	Yes
H14	Loneliness	Attachment	− 0.395**	Yes
H15	Autonomy	Autonomy	0.146**	No
H16	Personal growth	Achievement	0.241**	No

Hypothesis	SWB-5D scale	Cantril ladder	Spearman’s rho	Confirmed
H17	Sum score	Score	0.567**	Yes

*SWB-5D* subjective wellbeing-5 dimensions, *EQ-5D* EuroQol five-dimensional questionnaire, *VAS* Visual Analogue Scale, *ICECAP-A* ICEpop CAPability Adult

*p < 0.05, **p < 0.01, ***p < 0.001

Table 4 shows the results on the known-group hypotheses. The SEM was equalled to 0.09 which means that SWB-5D scores with a difference smaller than 0.09 can be attributed to measurement error, while bigger differences are likely due to actual differences between groups. All four (100%) hypotheses were confirmed (significant and larger than the SEM), which indicates a good validity.

**Table 4:** Results on known-group validity hypotheses for SWB-5D

Hypothesis	Known group	N	Mean rank score	Median	Range	*p* value	Confirmed
H18	EQ-5D VAS ≥ 65	816	453	20	11–25	< 0.0001	Yes
	EQ-5D VAS < 65	200	737	17	7–25		
H19	Cantril ladder ≥ 6.5	824	572	20	11–25	< 0.0001	
	Cantril ladder < 6.5	192	236	17	7–25		Yes
H20	No illness	475	447	20	8–25	< 0.0001	Yes
	Illness	541	563	19	7–25		
H21	Low	292	463	19.5	7–25	< 0.01	Yes
	Medium	433	521	20	11–25		
	High education	291	534	20	11–25		

*EQ-5D VAS* EuroQol five-dimensional questionnaire Visual Analogue Scale

### Interpretability

From all respondents, 2.65% scored no problems on all items of the SWB-5D. For the ICECAP-A, 6.79% of the patients scored the highest values on all items and for the EQ-5D 23.72%, indicating that the SWB-5D had the lowest ceiling effect.

## Discussion

The goal of this study was to assess the psychometric properties of a new outcome measure, the SWB-5D.

### Concurrent validity of the SWB-5D

Tested on a representative sample of the Dutch population, the concurrent validity showed mixed results. While the SWB-5D explained more than 35% of the variance in measurement of mobility, physical fitness, future perspective, contentment, social relations, and overall wellbeing, it explained less than 20% of the variance in measurements of “self-care,” “stability,” “autonomy,” and “achievement.” Most SWB-5D items showed theoretically sound statistical relationships with items from other scales. For example, physical independence loaded high on mobility (EQ-5D) and happiness was a predictor of scores on contentment (PH-17). Interestingly, the autonomy domain of the SWB-5D was not a predictor of the ICECAP-A autonomy domain, while “I live my life my own way” and “being completely independent” both might imply to do things the way you want to.

### Construct validity of the SWB-5D

Construct validity was explored with convergent and known-group validity. The SWB-5D indicated good convergent validity, since 12 of 15 predetermined hypothesis were confirmed (80%). The hypothesis testing whether physical independence (SWB-5D) correlate with the autonomy item (ICECAP-A) was not confirmed. Similarly to the result from the concurrent validity, the hypotheses that the items autonomy from the ICECAP-A and SWB-5D would correlate are not confirmed. Furthermore, good known-group validity was attained, since 4 of 4 hypotheses were confirmed (100%).

An explanation for the differences between the autonomy item from the ICECAP-A and SWB-5D could be that “I am able to be completely independent” (ICECAP-A) is interpreted as that you are able to do your usual activities independently [25], while “I live my life in my own way” (SWB-5D) might be interpreted as a way of life in which you make your own choices. Qualitative research is needed to further clarify this difference. Comparing opinions and interpretations of the items by respondents allows to investigate whether the intended non-tangible dimension is measured. Also the finding that the items’ physical independence of the SWB-5D and autonomy from the ICECAP-A do not correlate should be addressed in future qualitative research. While the use of a single item to measure a dimension has the advantage of reducing the burden for patients, it is likely to come with a disadvantage as well. Trying to capture a broad definition of health in only a few constructs with only one item per dimension may reduce reliability and validity, because items might be either formulated too broadly or fuzzy, or focus on different aspects of the same non-tangible dimension across different measures.

Correlations between the SWB-5D and the other tools were moderate which suggest that there is considerable overlap between the instruments, but also differences. These differences might reflect that additional aspects are measured with the SWB-5D which go beyond the existent measures [8]. This is supported by the fact that the SWB-5D explains a considerable amount of variance (56.7%) of the overall happiness measure (Cantril Ladder), which is higher than for the ICECAP-A and EQ-5D explaining, respectively, 52.1% and 49.1% of the variation in happiness (Online Resource 4).

### Interpretability

Compared to the EQ-5D and ICECAP-A, the SWB-5D showed more room to detect subtle changes in health. Only 2.65% of the respondents scored no problems on all items of the SWB-5D compared to the 6.79% of the ICECAP-A and 23.72% of the EQ-5D.

### Future research

To measure health comprehensively for the use in health-related economic evaluations, the NICE guideline [26] indicates that multiple instruments assess different constructs and can effectively complement each other. The Dutch guidelines for conducting economic evaluations in healthcare also specify that the ICECAP should be added when interventions aim to improve not only health, but wellbeing in terms of living situation, autonomy, and social interaction as well [27]. In line with this, the SWB-5D can be an additional measure for interventions for which mental and social aspects play an important role, e.g. that are more related to care than cure. However, adding questionnaires will lead to additional burden for the respondents, therefore, development of one new instrument in which all relevant pillars of health (physical, social and mental) are represented might be the ultimate goal. This study may add to this development. Another way forward may be the use of bolt-ons to existing measures that are frequently used such as the EQ-5D [28–30]. Several bolt-ons were identified already such as cognitive functioning, vision, hearing, relationships, and sleep [30] covering additional mental and social domains. Based on this study, we would suggest further research on adding mental and social domains, such as positive affect and personal growth.

### Strengths and limitations

A strength of this study was that a large sample representative of the general Dutch population participated. A second strength of this paper was the chosen validation scales. For convergent validity, the SWB-5D was compared to both a broadly used health measure (EQ-5D) and novel measures entailing other, broader, concepts such as the capability approach (ICECAP-A) and positive health (PH-17) which enhanced the interpretability of the results within the current health and wellbeing measurements landscape.

The findings of our study should be interpreted with care, taking the limitation that it was limited to the Dutch population into account. Further research should assess other quantitative psychometric properties, such as test–retest reliability. But, in line with other studies [31–33], our study highlights the importance that beyond a quantitative approach, it is essential to explore how the instrument succeeds in measuring outcomes that matter to patients, as well as patient’s understanding of the questions asked. For such purpose, qualitative methods can be applied such as, face validity, response process validity, cognitive interviewing, and comments to open-ended survey questions. Another factor that should be taken into account is that the coronavirus (COVID-19) pandemic was at the time of data collection a global health treat which might have negatively affected health and wellbeing outcomes. However, this is not expected to affect the assessment of psychometric properties.

Last, by developing the SWB-5D, the intention was to detect effects of (health) interventions in situations that are more care related than cure related [8]. Therefore, the SWB-5D, or an alternative broad health measurement, should also be tested and evaluated in these care situations to reveal the responsiveness of the instrument, i.e. whether the instrument is able to reflect the effects of (health) interventions in these patient groups.

## Conclusion

Adequate psychometric properties of outcome measures are vital for reliable use of the instrument in economic evaluations of interventions aimed at improving health and wellbeing. The present study showed good psychometric properties of the SWB-5D in a large representative Dutch sample. To conclude, this study is a step forwards in measuring health in a broad sense in health economic evaluations. This instrument demonstrates both overlap and differences with other measurement tools, indicating that the SWB-5D measures a distinct concept. The present study adds to the established literature that there is a need to qualitatively explore measures and for a novel default measure. Whether this could be the SWB-5D or another novel initiative, we should strive together for a more comprehensive health measure standard.

## Supplementary Information

Below is the link to the electronic supplementary material.

Supplementary file1 (PDF 82 KB)

Supplementary file2 (PDF 196 KB)

Supplementary file3 (PDF 129 KB)

Supplementary file4 (PDF 115 KB)
