# Supplementary material for: The assessment of psychometric properties for the subjective wellbeing-5 dimensions (SWB-5D) questionnaire in the general Dutch population
Source: Qual Life Res. 2022 Aug 20;32(1):237–45. doi: 10.1007/s11136-022-03234-8 (PMC9392428; doi:10.1007/s11136-022-03234-8)
Supplement: Supplementary file 1 — Supplementary file1 (PDF 82 KB) [file 11136_2022_3234_MOESM1_ESM.pdf]

*The assessment of psychometric properties for the Subjective Wellbeing-5 Dimensions (SWB-5D) questionnaire in a general Dutch population. Quality of Life Research.*

*H.N Haspels, M. de Vries, M.E. van den Akker-van Marle. Department of Biomedical Data Science, section Medical Decision Making Leiden University Medical Center, Leiden, The Netherlands. Email: vandenakker@lumc.nl.*

Online Resource 1: SWB-5D questionnaire

| | Completely disagree | Disagree | Neither agree nor disagree | Agree | Totally agree |
|---|---|---|---|---|---|
| My health state restricts my physical activities | o | o | o | o | o |
| During the past week, I felt happy | o | o | o | o | o |
| During the past week, I felt lonely | o | o | o | o | o |
| I live my life my own way | o | o | o | o | o |
| For me, life is a continuous process of learning, change and growth | o | o | o | o | o |
