# Supplementary material for: The assessment of psychometric properties for the subjective wellbeing-5 dimensions (SWB-5D) questionnaire in the general Dutch population
Source: Qual Life Res. 2022 Aug 20;32(1):237–45. doi: 10.1007/s11136-022-03234-8 (PMC9392428; doi:10.1007/s11136-022-03234-8)
Supplement: Supplementary file 2 — Supplementary file2 (PDF 196 KB) [file 11136_2022_3234_MOESM2_ESM.pdf]

The assessment of psychometric properties for the Subjective Wellbeing-5 Dimensions (SWB-5D) questionnaire in a general Dutch population. Quality of Life Research.
H.N Haspels, M. de Vries, M.E. van den Akker-van Marle. Department of Biomedical Data Science, section Medical Decision Making Leiden University Medical Center, Leiden, The Netherlands. Email: vandenakker@lumc.nl.

Online Resource 2: Overview overall psychometric testing, hypotheses construct validity and visualization on overlapping concepts tested.

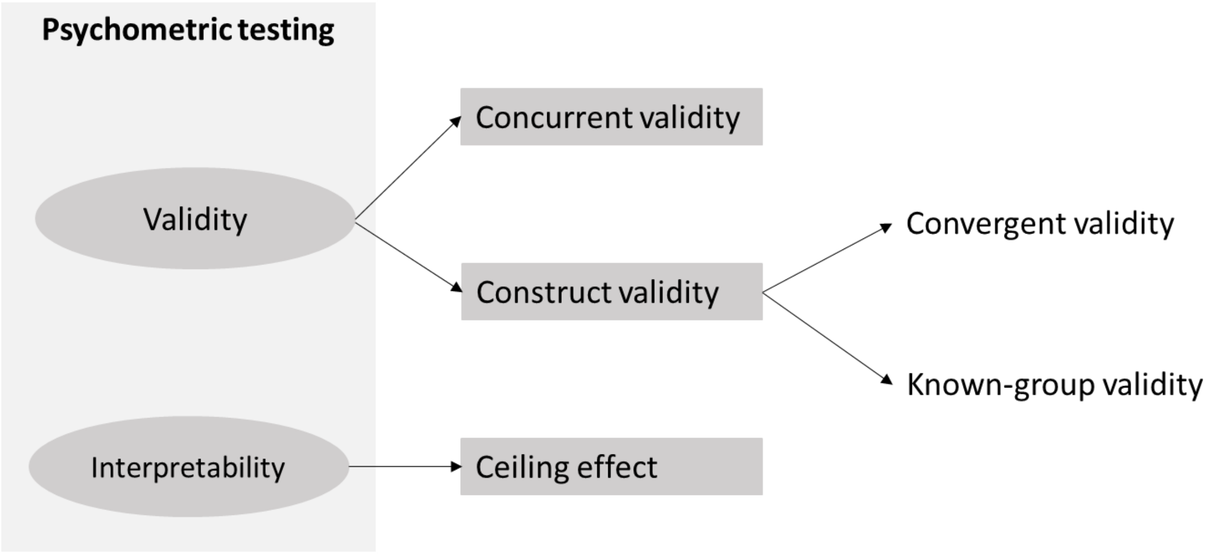

Convergent validity hypotheses

For all hypotheses we expected a significant moderate to high correlation (>0.35).

- H1: SWB-5D sum score and the EQ-5D utility score
- H2: SWB-5D physical independence subscale and the EQ-5D mobility subscale
- H3: SWB-5D physical independence subscale and the EQ-5D self-care subscale
- H4: SWB-5D physical independence subscale and the EQ-5D usual activity subscale
- H5: SWB-5D physical independence subscale and the EQ-5D pain/discomfort subscale
- H6: SWB-5D loneliness subscale and the EQ-5D anxiety subscale
- H7: SWB-5D happiness subscale and the EQ-5D anxiety subscale
- H8: SWB-5D sum score and the EQ-VAS
- H9: SWB-5D sum score and the ICECAP-A capability score
- H10: SWB-5D physical independence subscale and the ICECAP-A autonomy subscale
- H11: SWB-5D happiness subscale and the ICECAP-A enjoyment subscale
- H12: SWB-5D happiness subscale and the ICECAP-A attachment subscale
- H13: SWB-5D loneliness subscale and the ICECAP-A enjoyment subscale
- H14: SWB-5D loneliness subscale and the ICECAP-A attachment subscale
- H15: SWB-5D autonomy subscale and the ICECAP-A autonomy subscale
- H16: SWB-5D personal growth subscale and the ICECAP-A achievement subscale
- H17: SWB-5D sum score and Cantril Ladder score

Known group hypotheses

For all hypotheses we expected a significant differences and a difference greater than the SEM.

- H18: There will be a difference in SWB-5D scores between higher and lower EQ-5D VAS scores. Higher self-reported health will lead to higher SWB-5D scores.
- H19: There will be a difference in SWB-5D scores between higher and lower Cantril ladder scores. Higher self-reported happiness will lead to higher SWB-5D scores.
- H20: There will be a difference in SWB-5D scores between being ill or not. No illness will lead to higher SWB-5D scores.
- H21: There will be a difference in SWB-5D scores between the three education levels. Higher education will lead to higher SWB-5D scores.

		SWB-5D					
		Physical independence	Happiness	Loneliness	Autonomy	Personal growth	Sum score
EQ-5D	Mobility	H2					
	Self-care	H3					
	Usual activities	H4					
	Pain/Discomfort	H5					
	Anxiety/Depression		H7	H6			
	Utility (weighted sum) score						H1
EQ-VAS	Utility score						H8
ICECAP-A	Stability						
	Attachment		H12	H14			
	Autonomy	H10			H15		
	Achievement					H16	
	Enjoyment		H11	H13			

	Sum score	
	Capability (weighted sum) score	H9
Cantril Ladder	Score	H17
