# Supplementary material for: The assessment of psychometric properties for the subjective wellbeing-5 dimensions (SWB-5D) questionnaire in the general Dutch population
Source: Qual Life Res. 2022 Aug 20;32(1):237–45. doi: 10.1007/s11136-022-03234-8 (PMC9392428; doi:10.1007/s11136-022-03234-8)
Supplement: Supplementary file 3 — Supplementary file3 (PDF 129 KB) [file 11136_2022_3234_MOESM3_ESM.pdf]

The assessment of psychometric properties for the Subjective Wellbeing-5 Dimensions (SWB-5D) questionnaire in a general Dutch population. Quality of Life Research.

H.N Haspels, M. de Vries, M.E. van den Akker-van Marle. Department of Biomedical Data Science, section Medical Decision Making Leiden University Medical Center, Leiden, The Netherlands. Email: vandenakker@lumc.nl.

Online Resource 3: Correlation matrix between the SWB-5D, EQ-5D and ICECAP-A

		SWB-5D				
		Physical independence	Happiness	Loneliness	Autonomy	Personal growth
EQ-5D	Mobility	0,62**	0,171**	0,089**	0,061	0,134**
	Self-care	0,331**	0,141**	0,086**	0,045	0,046
	Usual activities	0,484**	0,328**	0,244**	0,120**	0,097**
	Pain/discomfort	0,444**	0,174**	0,118**	0,003	0,074*
	Anxiety/ depression	0,099**	0,443**	0,477**	0,202**	0,075*
ICECAP-A	Stability	0,167**	0,349**	0,285**	0,231**	0,112**
	Attachment	0,110**	0,430**	0,395**	0,182**	0,179**
	Autonomy	0,220**	0,222**	0,225**	0,146**	0,092**
	Achievement	0,256**	0,383**	0,282**	0,186**	0,241**
	Enjoyment	0,182**	0,511**	0,396**	0,243**	0,220**

Abbreviations: SWB-5D; Subjective wellbeing 5 dimensions, EQ-5D; EuroQol five-dimensional questionnaire, ICECAP-A; ICEpop CAPability Adult

** Correlation is significant at the 0.01 level (2-tailed).

* Correlation is significant at the 0.05 level (2-tailed).
