# Supplementary material for: The assessment of psychometric properties for the subjective wellbeing-5 dimensions (SWB-5D) questionnaire in the general Dutch population
Source: Qual Life Res. 2022 Aug 20;32(1):237–45. doi: 10.1007/s11136-022-03234-8 (PMC9392428; doi:10.1007/s11136-022-03234-8)
Supplement: Supplementary file 4 — Supplementary file4 (PDF 115 KB) [file 11136_2022_3234_MOESM4_ESM.pdf]

The assessment of psychometric properties for the Subjective Wellbeing-5 Dimensions (SWB-5D) questionnaire in a general Dutch population. Quality of Life Research.
H.N Haspels, M. de Vries, M.E. van den Akker-van Marle. Department of Biomedical Data Science, section Medical Decision Making Leiden University Medical Center, Leiden, The Netherlands. Email: vandenakker@lumc.nl.

Online Resource 4: Correlation matrix between the SWB-5D, EQ-5D, ICECAP-A, Cantril Ladder and EQ-VAS.

	SWB-5D	EQ-5D	ICECAP-A
Cantril Ladder	0.567**	0.491**	0.521**
EQ-VAS	0.529**	0.529**	0.445**

Abbreviations: SWB-5D; Subjective wellbeing 5 dimensions, EQ-5D; EuroQol five-dimensional questionnaire, VAS; Visual Analogue Scale, ICECAP-A; ICEpop CAPability Adult

** Correlation is significant at the 0.01 level (2-tailed).
